# Double-blind, 12 month follow-up, placebo-controlled trial of mifepristone on cognition in alcoholics: the MIFCOG trial protocol

**DOI:** 10.1186/s12888-016-0757-1

**Published:** 2016-02-24

**Authors:** Kim Donoghue, Abigail Rose, Simon Coulton, Joanna Milward, Kylie Reed, Colin Drummond, Hilary Little

**Affiliations:** Addictions Department, National Addiction Centre, Institute of Psychiatry, King’s College London, 4 Windsor Walk, London, SE5 8BB UK; Department of Psychological Sciences, University of Liverpool, 2.32, Eleanor Rathbone Building, Bedford Street South, Liverpool, L69 7ZA UK; Centre for Health Service Studies, University of Kent, Canterbury, Kent CT2 7NF UK

**Keywords:** Alcohol dependence, Memory, Cognitive function, Depression, Cortisol, Glucocorticoid Type II receptor, Mifepristone

## Abstract

**Background:**

Increased levels of cortisol during acute alcohol withdrawal have been linked to cognitive deficits and depression. Preclinical research found that the glucocorticoid Type II receptor antagonist, mifepristone, prevented some of the neurotoxic effects of withdrawal and memory loss. Clinical trials have shown mifepristone effective in the treatment of depression. This study aims to examine the extent to which the glucocorticoid Type II receptor antagonist, mifepristone, when given to alcohol dependent males during the acute phase of alcohol withdrawal, will protect against the subsequent memory loss and depressive symptoms during abstinence from alcohol.

**Methods/Design:**

The study is a Phase 4 therapeutic use, “Proof of Concept” trial. The trial is a double-blind randomised controlled clinical trial of mifepristone versus inactive placebo. The trial aims to recruit 120 participants referred for an inpatient alcohol detoxification from community alcohol teams, who meet the inclusion criteria; 1) Male, 2) Aged 18–60 inclusive, 3) alcohol dependent for 5 or more years. A screening appointment will take place prior to admission to inpatient alcohol treatment units to ensure that the individual is suitable for inclusion in the trial in accordance with the inclusion and exclusion criteria. On admission participants are randomised to receive 600 mg a day of mifepristone (200 mg morning, afternoon and evening) for 7 days and 400 mg for the subsequent 7 days (200 mg morning and evening) or the equivalent number of placebo tablets for 14 days. Participants will remain in the trial for 4 weeks (at least 2 weeks as an inpatient) and will be followed up at 3, 6 and 12 months post randomisation. Primary outcome measures are cognitive function at week 3 and 4 after cessation of drinking and symptoms of depression over the 4 weeks after cession of drinking, measured using the Cambridge Neuropsychological Test Automated battery and Beck Depression Inventory, respectively. Secondary outcome measures are severity of the acute phase of alcohol withdrawal, alcohol craving, symptoms of protracted withdrawal and maintenance of abstinence and levels of relapse drinking at follow-up.

**Discussion:**

The current trial will provide evidence concerning the role of glucocorticoid Type II receptor activation in cognitive function and depression during acute alcohol withdrawal and the efficacy of treatment with mifepristone.

**Trial registration:**

ISRCTN: ISRCTN54001953, Registered 29th September 2011.

## Background

Cognitive deficits are seen in 50 to 80 % of those dependent on alcohol [1] currently there is no effective treatment. The cognitive functions particularly affected in alcohol dependence are learning new skills, visuospatial ability, executive function (planning, capacity for abstraction and effortful processing), and aspects of attention, whilst verbal skills usually remain intact [2–6]. The cognitive deficits not only affect the quality of life of those dependent on alcohol and the amount of health care they need, but also have a detrimental effect on ability to benefit from treatment programmes [4, 7, 8]. Depression is a major symptom in many people who are dependent on alcohol, and is associated with greater tendency to relapse drinking [9]. Most depressive symptoms in this population are secondary to the alcohol dependence and gradually resolve spontaneously. Despite this, improvement in symptomatology could provide faster and more complete recovery and would aid abstinence.

The majority of those dependent on alcohol undergo frequent episodes of withdrawal and resumption of drinking, and clinical evidence indicates that “kindling” occurs, as a higher number of withdrawal episodes are associated with greater severity of withdrawal, including greater memory impairment [10]. Schandler et al. [11] found visuospatial learning was worse in those dependent on alcohol during abstinence than in those who were still intoxicated. People who experienced more alcohol withdrawal episodes have been found to have greater subsequent memory deficits [12]. Preclinical data also demonstrated that withdrawal is an important factor in causing the memory deficits. Memory and learning deficits were found in rats in a variety of tests after alcohol withdrawal, but not during alcohol intake [13, 14]. These data suggest that the neurotoxicity that is known to occur during acute alcohol withdrawal may play a contributory role in the subsequent cognitive deficits so frequent in those who are dependent on alcohol [15].

Benzodiazepine drugs are normally prescribed during alcohol detoxification to reduce the overt symptoms of alcohol withdrawal (tremor, and potentially fatal convulsions) but these drugs do not prevent the longer term consequences of cognitive deficits, depression, alcohol craving and relapse back into alcohol drinking [4, 12, 16]. However, drugs that act at different target sites from that of the benzodiazepines could have prolonged beneficial effects. The acute alcohol withdrawal phase therefore offers a potential therapeutic window for intervention that has previously been little explored.

There is a high level of release of glucocorticoids (cortisol in humans, corticosterone in rodents) from the adrenals during the acute alcohol withdrawal period. High glucocorticoid levels are well established to cause memory loss in both humans and animals [17]. High circulating concentrations of cortisol and corticosterone activate the Type II glucocorticoid receptor. These hormones increase the neurotoxic effects of *N*-Methyl-D-aspartate (NMDA) and other excitatory amino acids [18]. An important link between cortisol and memory loss was found by Errico et al. [19] who showed the severity of cognitive deficits in alcohol-dependent persons was correlated not only with a greater number of withdrawal episodes but also with higher cortisol levels during acute withdrawal. There is also evidence relating depressive symptoms and cognitive deficits [20]. Although the direction of any potential cause/effect relationship is not yet fully understood, glucocorticoids have been implicated in both problems [21].

Mifepristone is an antagonist at the Type II glucocorticoid receptor. Administration of mifepristone to mice during the acute phase of alcohol withdrawal was found to reduce the subsequent memory loss [22]. Parallel studies, using an organotypic culture model of alcohol withdrawal, showed that high glucocorticoid concentrations of glucocorticoid increased the neurotoxic consequences of alcohol withdrawal. Application of the Type II glucocorticoid receptor antagonist, mifepristone, prevented this neurotoxicity [23]. In preclinical studies, mifepristone also reduced the behavioural signs of acute alcohol withdrawal [23, 24]. Voluntary alcohol consumption in rodents, and increases in this caused by mild stress, were reduced by administration of mifepristone [25, 26]. Fahlke and colleagues [27, 28] showed that administration of corticosterone increased voluntary alcohol drinking in rats, while reduction in glucocorticoid synthesis, or adrenalectomy, decreased alcohol intake. Importantly for the current study, Vendroluscolo et al. [29] showed that mifepristone reduced the increase in operant responding for alcohol seen in alcohol-dependent rats, without affecting responding in rats not dependent on alcohol. In a recent clinical trial, mifepristone was administered at 600 mg daily for 1 week to alcohol-dependent individuals who continued to drink alcohol for the first 4 days of the treatment. Reductions in alcohol-cue craving and in self-reported alcohol consumption were seen after 1 week of treatment, and in the latter at 1 week post treatment [30].

Clinical studies have found mifepristone also has a beneficial effect in depression [31–35] with a more rapid action than conventional antidepressants [34]. A clinical pilot study found mifepristone alleviated both the cognitive deficits and the depressive symptoms in bipolar depression [35]. However, a subsequent larger study by the same research group found only cognitive deficits were alleviated, specifically spatial working memory [36]. There are no published reports of glucocorticoid antagonist trials for treatment of depression in alcohol-dependent individuals, although in some alcoholics, conventional antidepressant drugs both alleviated depressive symptoms and decreased relapse drinking [37].

### Aims of the study

The current study aims to examine the extent to which the Type II glucocorticoid receptor antagonist, mifepristone, when given to alcohol-dependent persons during the acute phase of alcohol withdrawal, will protect against the subsequent memory loss and depressive symptoms during abstinence from alcohol. The results will provide information about the extent of the contribution of glucocorticoids to these problems, which will aid future development of treatment for alcohol dependence.

### Objectives

- To compare effects of mifepristone and placebo in preventing memory loss and depressive symptomatology in alcohol-dependent persons undergoing alcohol detoxification, using the Cambridge Neuropsychological Test Automated battery (CANTAB) and Beck Depression Inventory (BDI-II) [38], respectively.
- To compare effects of mifepristone and placebo on the severity of the acute phase of alcohol withdrawal, alcohol craving and the symptoms of protracted withdrawal.
- To compare effectiveness of mifepristone and placebo in maintaining abstinence from alcohol and levels of relapse drinking at 3, 6 and 12 months follow-up from cessation of alcohol consumption.

## Methods/Design

The design of the current study is a double-blind randomised controlled clinical trial of mifepristone and inactive placebo. The study is a Phase 4 therapeutic use, “Proof of Concept” trial. Ethical approval has been granted by the London–Dulwich Research Ethics Committee (reference: 10/H0808/7) and complies with UK and European Good Clinical Practice for medicinal trials guidelines. Participants will be recruited from Community Alcohol Teams serving 5 inpatient alcohol treatment units in 3 regions of England (South London, Sussex, Kent, Hull and Barnsley). Participants will be given mifepristone/placebo for 2 weeks while they are in patients undergoing detoxification from alcohol. They will then remain in the trial for a further 2 weeks undergoing cognitive testing and questionnaires, with follow-up contact at 3, 6 and 12 months subsequent to alcohol cessation.

### Hypotheses

- Treatment with mifepristone will result in improved memory and/or depressive symptomatology outcomes compared with placebo.
- Relapse drinking rates will be reduced in those treated with mifepristone compared with placebo.
- Activation of Type II glucocorticoid receptors during the acute phase of alcohol withdrawal is causally involved in the subsequent cognitive deficits, symptoms of depression and/or relapse drinking following acute cessation of alcohol consumption.

### Inclusion criteria

1. Diagnosis of alcohol dependence by DSM-IV for 5 years or more
2. Male
3. Aged between 18 and 60 years (inclusive)
4. Willingness to provide informed consent.

### Exclusion criteria

1. Clinical diagnosis of a neuroendocrine disorder
2. Liver damage, determined at screening by alanine aminotransferase (ALT) activity of more than 2.5 times the normal range
3. Renal dysfunction, as determined by creatinine levels over 150 μmol/L in plasma samples taken prior to admission to the Alcohol Unit
4. Documented evidence of a psychotic disorder
5. Severe brain damage or severe mental impairment
6. Diagnosis of severe physical illness that would preclude participation (e.g. terminal illness),
7. Documented evidence of current dependence on a substance other than alcohol or nicotine.
8. Inability to understand sufficient English to understand the information needed for the cognitive testing
9. Wernicke-Korsakoff syndromes
10. Porphyria
11. Severe asthma uncontrolled by therapy
12. Cardiac disorders i.e. myocardial infarction, congestive cardiac failure or cardiomyopathy
13. Persistent high blood pressure (over 160 mmHg systolic and/or 100 mmHg diastolic on both of two readings at initial screening session
14. Medical history of diabetes
15. A known allergy to mifepristone
16. Owing to potential interactions with mifepristone, participants taking the following drugs will be excluded unless a suitable alternative can be prescribed: ketoconazole, itraconazole, metronodazole, miconazole, erythromycin, clarithromycin, rifampin, rifabutin, norfloxacin, nelfinavir, ritonavir, saquinavir, zafirlukast, fluvoxamine, quinine, phenytoin, phenobarbital, primadone, carbamazepine, amiodarone, warfarin, corticosteroids or St John’s Wort. Consumption of grapefruit juice is also contraindicated during the mifepristone treatment.

### Study entry

Prospective participants who are referred for an admission to an alcohol inpatient unit for alcohol detoxification will be identified by the community alcohol teams. If the person is willing to hear more about the research, contact details will be passed to the research team. Written informed consent to complete a preliminary screening appointment will be taken. Eligibility to take part in the research will be checked at a preliminary screening appointment and included administration of the Mini-Mental Sate Examination (MMSE) [39] to screen for any severe cognitive impairment. The Composite International Diagnostic Interview (CIDI) [40] will be administered to demonstrate alcohol dependence of at least 5 years of the patients prior to detoxification according to DSM-IV diagnostic criteria. Plasma alanine aminotransferase (ALT) and creatinine will be measured for indication of liver damage and impaired renal function, respectively. Urine will be tested for evidence of porphyria. If the participant is eligible, consent to take part in the study will be obtained prior to or on admission to the alcohol unit by a clinical member of the research team or a delegated physician. All participants will be advised not to consume grapefruit juice during the trial as this is contraindicated with mifepristone.

At the preliminary screening appointment participants will be asked to complete questionnaires to help establish patterns of alcohol consumption. This includes a measure of the severity of alcohol dependence using the Severity of Alcohol Dependence Questionnaire (SADQ) [41], current craving for alcohol using the Alcohol Urge Questionnaire (AUQ) [42], problems experienced due to alcohol using the Alcohol Problems Questionnaire (APQ) [43], and a retrospective report of the quantity of alcohol consume over the previous 90 days collected using the time-line follow-back (TLFB) [44]. In addition, the Fagerstrom questionnaire [45] will be used to record the amount of smoking and level of nicotine dependence and symptoms of depression will be measured using the Beck Depression Inventory (BDI-II) [46].

### Randomisation and blinding

Randomisation will be carried out after consent is gained for participation in the main trial for those participants who are eligible. A remote randomisation procedure will be used through an online system based at the King’s Clinical Trials Unit at the Institute of Psychiatry, King’s College London. This involves allocation of unique identification numbers for each participant. The minimisation method will be used for the allocation that involves stratification by severity of alcohol dependence (SADQ score of over 40 or under 40), site and age (18 to 29, 30 to 39, 40 to 49 or 50 to 60). These variables are known to be potential confounders with the observed outcome and are collected at preliminary screening.

As this is a multi-centre study and allocation is blind, those conducting the randomisation will not be in a position to subvert the allocation sequence. The blister packs contain 7 tablets and will be supplied in individual cartons with a label on the front face of the carton bearing a unique kit identification number (Kit ID). The Kit ID is pre-printed on the label by the packaging company. Participants will be allocated Kit IDs according to the remote randomisation procedure. Each carton sent to the Pharmacy by the manufacturing company will be accompanied by an individual sealed code-break envelope in case of emergency unblinding. Participants, researchers and ward staff will be blind to which treatment each patient is given.

### Pharmacological treatment

The duration of treatment with mifepristone/placebo is 2 weeks, while participants are an inpatients in the alcohol treatment units. The daily dose will be three 200 mg tablets a day (morning, afternoon and evening) for 7 days followed by two 200 mg tablets a day (morning and evening). These doses are based on previous studies of the effectiveness and toxicity of mifepristone.

The participants will receive normal clinical care while on the unit, and concurrent medical and psychiatric disorders will be treated as required. This includes prescription of a benzodiazepine (normally chlordiazepoxide) to protect against the tremor and convulsive aspects of acute alcohol withdrawal. Benzodiazepine treatment is normally given to patients during the first 7 to 10 days of detoxification, as required, and this treatment will not be altered during the trial. All prescriptions of any drug treatment given to trial participants will be recorded during the trial.

All participants will receive the standard care provided by the participating alcohol treatment services. This includes access to all the facilities provided by the services, including psychotherapy, pharmacotherapy, inpatient and residential rehabilitation. These services are normally provided for up to 3 months per episode of care. Participants who relapse or seek further treatment will be offered additional care, in keeping with standard clinical policies.

### Assessment of efficacy

**Fig. 1:**
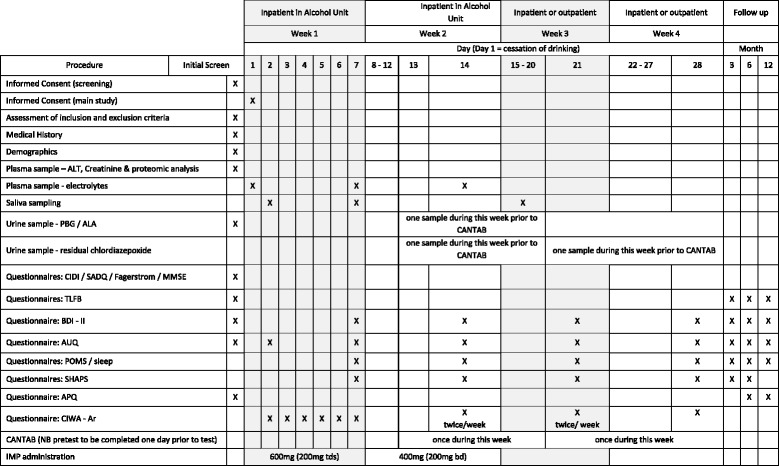
Trial procedure timeline

Figure 1 presents an overview of the trial protocol.

### Primary outcome measures

Cognitive ability will be measured using the CANTAB. The CANTAB has been used extensively in research and previous studies have been published that have used the CANTAB test battery in patients with depression and patients with alcohol dependence [47–52]. The CANTAB testing will compare mifepristone and placebo groups concurrently. Cognitive function measurement prior to mifepristone/placebo would not be valid owing to intoxication. A pretest will be carried out on the day before the first CANTAB testing, composed of the Motor Screening task and the Reaction time task. These two tests are designed to assess basic motor and visual skills and determine whether participants are capable of being tested on CANTAB. The CANTAB tests that will be used at the two cognition testing times (between days 13 and 20 and 21 and 28) are: visuospatial ability by Reaction Time, Pattern Recognition, Matching to Sample Visual Search, Spatial Recognition memory and Paired Associates tasks; sustained attention by the Rapid Visual Processing task; and executive function by Intra- and Extra-Dimensional Shifting, Spatial Working Memory and Stockings of Cambridge tasks.

The above CANTAB test times will enable examination of cognitive testing administration of chlordiazepoxide ceases, as benzodiazepines are well established to affect memory and learning. Urine samples taken on the day of CANTAB testing will be screened for the presence of residual chlordiazepoxide or its active metabolites, which may affect cognitive performance [53].

The BDI-II [46] will be used to record symptoms of depression throughout the trial. It will be administered at every 7 days during the 4 weeks the participant is in the trial and at 3, 6 and 12 months follow-up from cessation of drinking. The BDI-II will be administered on the day of the CANTAB testing so that the results can be used as a variable in the measure of cognitive function.

### Secondary outcomes

Severity of the overall syndrome of acute alcohol withdrawal will be monitored using the Clinical Institute Withdrawal Assessment for Alcohol (CIWA-Ar) [54]. This questionnaire will be carried out once per day for the first week, starting 24 h after drinking cessation, as in normal clinical care, then twice every 7 days (±2 days) for the following 2 weeks, and once during week 4.

The AUQ will be administered every 7 days, with the first administered 24 h after drinking cessation, to measure alcohol craving. It will be administered at 3, 6 and 12 months follow-up.

Symptoms of protracted alcohol withdrawal include anxiety, fatigue, irritability, hyperarousal and depression [18]. The sleep questionnaire evaluates sleep disturbances that are a common problem during the early weeks of abstinence. This questionnaire will be carried out every 7 days (±2 days), starting 1 week after cessation of drinking and at 3, 6 and 12 months follow-up. The Profile of mood states (POMS) [55] measures current mood covering eight factors: anxiety, fatigue, depression, anger, vigour, confusion, friendliness and elation. The POMS will be carried out every 7 days (±2 days), starting 1 week after drinking cessation and at 3, 6 and 12 month follow-up. The Snaith Hamilton Pleasure Scale (SHAPS) [56] is designed to assess an individual’s ability to experience pleasure. The SHAPS will be carried out on days 7, 14, 21, 28 (±2 days) and at 3, 6 and 12 month follow-up after cessation of drinking.

A maximum of 21 saliva samples will be collected from each participant during the trial. Seven saliva samples will be collected during the course of the day on three different occasions (day 2, 7 and 20 ± 2 days after cessation of alcohol drinking). Samples will be collected in prepared tubes and were analysed for cortisol concentrations to determine the cortisol levels prior to and following mifepristone or placebo treatment. Saliva samples will only be collected while participants are in-patients; saliva sample collection will not be taken from participants who are out-patients.

Maintenance of abstinence from alcohol and levels of relapse drinking will be measured using the TLFB, administered at preliminary screening and at 3, 6 and 12 months follow-up after cessation of drinking.

### Assessment of safety

The procedures involved in this research are unlikely to cause significant discomfort or distress to participants. All questionnaire measures and research procedures have been widely validated and used in previous research without causing any problems to participants. Mifepristone has a good safety record and has been used clinically for over 15 years for other applications. However should any distress or discomfort occur with any of the procedures trained medical and nursing staff will be available on a 24 h basis to provide help and support. All staff involved in the care of trial participants will receive training on the conduct of the trial protocol. In particular training will be provided on the detection and management of expected and unexpected adverse drug events.

The collection and reporting of Adverse Events will start following the first dose of the trial medication and will continue until the last dose of the trial medication. Reporting of Serious Adverse Events, Serious Adverse Reactions and Suspected Unexpected Serious Adverse Reactions will continue until 14 days after stopping the trial medication. All concomitant medications will be recorded until 14 days after stopping the trial medication.

Daily assessments by the clinical team to monitor for signs of nausea or fatigue that might indicate adrenal insufficiency will be made. Analysis of the plasma samples that will be taken on days 1, 7 and 14 of the mifepristone/placebo administration will ensure that the electrolyte levels remain within normal limits (sodium 135–145, potassium 3.5–5.3, chloride 95–105, urea 3.3–6.7, creatinine 40–120, all units micromoles per litre). Blood pressure checks were carried out daily during the acute withdrawal phase (10–14 days) as part of the normal clinical routine, and on day 14 if not measured on that day as part of normal clinical care. In the event of a participant self-discharging from the unit before the course of mifepristone is completed, the mifepristone/placebo treatment will be stopped and the participant will be withdrawn from the trial.

Following recruitment of the first 20 participants the Data Monitoring Committee will carry out a detailed examination of the safely data to ensure that there no problems have arisen with regard to drug safety.

### Honorarium

A £10 voucher for a local store that cannot be used to buy alcohol, will be given after completion of preliminary screening, on each of the 2 days of completion of the CANTAB testing plus a further voucher on completion of the follow-up contacts.

### Sample size

As this is a Proof of Concept study, previous data on the target population was not available. However, previously published studies using the CANTAB test battery in depressed patients have shown effect sizes of 0.5, 0.8, 0.76 [48], 0.496, 0.5 [51], 0.897 [49] and, 0.7 for spatial working memory in the elderly [50]. Similar values were obtained studying effects of acute alcohol withdrawal using an early version of the CANTAB [52]. We estimated the numbers needed in each group for a variety of effect size estimates based on a power of 80 % and alpha of 0.05. For an effect size difference of 0.8 25 subjects per group would be required and this increased to 32 for an effect size difference of 0.7 and 63 if the difference was 0.5. As a proof of principle study we erred on the side of caution and aimed to detect a small to medium effect size of 0.5 and this required 120 subjects participating in the study.

### Statistical analysis

The null hypothesis is that there will be no differences in the results from the cognitive testing between participants who received mifepristone and those who received the placebo. Primary data analysis will be by analysis of variance (ANOVA) on the CANTAB results and Beck depression scale scores for mifepristone and placebo groups, with post hoc tests for variables likely to affect these results. Analysis will be carried out both on a per protocol basis and an intention to treat basis.

A repeated measures analysis of variance using treatment type as a between-subject factor (two levels: mifepristone and placebo) and number of times the scale is applied as the within-subjects factor (five levels) will be carried out on the Beck depression scale results. This analysis will examine changes in level of depressive symptoms over the 4 weeks of the study. The analysis on the results from the BDI-II will compare the depressive symptoms prior to mifepristone/placebo with the scores during the four test weeks. This will provide information about whether or not mifepristone alters the progression of such symptoms and enable effect sizes to be calculated for such multiple comparisons in a full trial.

#### Cognitive performance

Reliable estimates of potential effects and variances will be obtained which will enable such repeated analysis in future, full clinical, trials (repeated measures analyses will not possible in this initial Proof of Concept study, as there will be insufficient information on between and within group variances).

#### Post hoc analysis

Although participants in the two treatment groups will be balanced, potential confounds will be examined as covariates in the post hoc ANCOVA analysis. Covariates will include: severity of withdrawal symptoms (measured by the CIWA-Ar, which primarily examines autonomic symptoms, and the AUQ which measures alcohol craving); presence/absence of depressive symptoms; smoking/non-smoking, chlordiazepoxide dosage; other medication prescribed after detoxification; co-morbid psychiatric disorders other than depression, other medication and age.

### Data management

Data will be anonymised and be stored by secure means. Electronic data will be stored on password protected computers or laptops. Hard copy data will be stored in locked filing cabinets in lockable offices in buildings with swipe access and security presence. All data will be entered into a computerised data entry system at King’s College London. At the end of the study arrangements will be made with the appropriate data repository service for transfer and preservation of the data in accordance with the principles agreed by the NHS and the MRC for the preservation and sharing of clinical data.

The results from the CANTAB cognition testing have to be entered directly on to a laptop as this is an integral part of this testing. Immediately at the end of each test session, the data entered on the laptop will be encrypted using a file encryption key to which only the research team will have access. No personal data will be entered on the laptop; the data will be stored under code numbers so that individual participants cannot be identified. As soon as possible after the end of a test session the data will be transferred to a secure server at King’s College London and the data erased from the laptop.

In order that contact can be made for the follow-up interviews, identifiable patient information will be accessed by the research team from the medical notes, with the patient’s consent. No personal information will be stored with the trial data.

Arrangements will be made for a copy of all the original data to be “locked” electronically prior to the analysis and kept at King’s College London in a secure location so that a record will be available of all the original results. The data will also be stored on the computer data system for archiving at the end of the study.

The investigators and Institution will permit trial-related monitoring, audits, Research Ethics Committee and Research and Development department review and regulatory inspections by providing direct access to source data and other documents (results of cognitive testing, depression scales, blood, saliva and urine test reports) and data analysis results.

### Monitoring

An independent Steering Committee has been set up, chaired by Professor Nicol Ferrier, Newcastle University. Members: Professor Anne Lingford Hughes, Imperial College and Professor Theodora Duka, Sussex University. The Medical Research Council has approved this membership. A Data Monitoring Committee has been set up and includes a statistician who is not otherwise involved in the project. A project management group will meet regularly to monitor the progress of the trial. This group will include the Chief Investigator, the Principal Co-Investigators, the researchers and clinicians and user representative. The trial will also be monitored in accordance with the King’s Health Partners Clinical Trials Office Quality Policy.

## Discussion

Preclinical trials have suggested that Type II glucocorticoid receptor activation during acute alcohol withdrawal is related to impairment of cognitive function. Treatment with mifepristone, a Type II glucocorticoid receptor antagonist, may protect against this. Clinical studies have shown that mifepristone has a beneficial effect in depression. The MIFCOG study is the first clinical trial to examine the role of glucocorticoid receptor activation in cognitive function and depression during protracted alcohol withdrawal, and the efficacy of treatment with mifepristone.

### Trial status

Recruitment for the trial began in September 2012 and was completed in May 2015. A total of 26 participants were recruited between September 2012 and September 2014. The trial experienced several challenges in recruitment over this 2 year period including retendering of NHS addiction services and the closure of NHS specialist addiction units at participating research sites. In September 2014 the decision was made to recruit participants who were completing their detoxification as an outpatients as well as an inpatients, to increase the number of potential participants. This decision was made following advice from the Trial Steering Committee and the Data Monitoring Committee and the results of the safety report following recruitment of the first 20 participants. Approval from the MHRA and local Research Ethics Committee to recruit participants completing their detoxification as outpatients was granted in November 2014. The study protocol was largely unchanged for outpatient recruitment, with the following exceptions;

1. Saliva sample collection was removed as it was not possible to gain reliable waking samples if participants were not an inpatient.
2. CIWA assessments were only completed for days 2–5 as there were insufficient staffing resources to complete these assessments at a weekend.
3. Plasma samples taken on days 7 and 14 were taken on days 7 and 14 +/− 2 days to allow for times when they fell on a weekend.

Following approval to recruit participants completing their detoxification as outpatients as well as those completing a detox as inpatients only 1 further participant was recruited. Completion of the final 12 month follow-up will be completed in May 2016.
